# Development of a strategy to functionalize a dextrin-based hydrogel for animal cell cultures using a starch-binding module fused to RGD sequence

**DOI:** 10.1186/1472-6750-8-78

**Published:** 2008-10-14

**Authors:** Susana M Moreira, Fábia K Andrade, Lucíla Domingues, Miguel Gama

**Affiliations:** 1 IBB, Institute for Biotechnology and Bioengineering, Centre of Biological Engineering, Universidade do Minho, Campus de Gualtar, 4710-057, Braga, Portugal

## Abstract

**Background:**

Several approaches can be used to functionalize biomaterials, such as hydrogels, for biomedical applications. One of the molecules often used to improve cells adhesion is the peptide Arg-Gly-Asp (RGD). The RGD sequence, present in several proteins from the extra-cellular matrix (ECM), is a ligand for integrin-mediated cell adhesion; this sequence was recognized as a major functional group responsible for cellular adhesion. In this work a bi-functional recombinant protein, containing a starch binding module (SBM) and RGD sequence was used to functionalize a dextrin-based hydrogel. The SBM, which belongs to an α-amylase from *Bacillus *sp. TS-23, has starch (and dextrin, depolymerized starch) affinity, acting as a binding molecule to adsorb the RGD sequence to the hydrogel surface.

**Results:**

The recombinant proteins SBM and RGD-SBM were cloned, expressed, purified and tested in *in vitro *assays. The evaluation of cell attachment, spreading and proliferation on the dextrin-based hydrogel surface activated with recombinant proteins were performed using mouse embryo fibroblasts 3T3. A polystyrene cell culture plate was used as control. The results showed that the RGD-SBM recombinant protein improved, by more than 30%, the adhesion of fibroblasts to dextrin-based hydrogel. In fact, cell spreading on the hydrogel surface was observed only in the presence of the RGD-SBM.

**Conclusion:**

The fusion protein RGD-SBM provides an efficient way to functionalize the dextrin-based hydrogel. Many proteins in nature that hold a RGD sequence are not cell adhesive, probably due to the conformation/accessibility of the peptide. We therefore emphasise the successful expression of a bi-functional protein with potential for different applications.

## Background

Hydrogels are a class of water-swollen polymeric materials, capable of maintaining a distinct three-dimensional structure [1,2], which can be used as scaffolds in tissue engineering, as wound dressing, and drug delivery systems, among other applications [3]. Several approaches have been developed to produce hydrogels from different synthetic and natural polymers [4]. Among them, the starch-based hydrogels have appealing characteristics in the perspective of biomedical application. They are biocompatible, have convenient degradation kinetics and release profiles and also present appropriate mechanical properties [5-7]. Despite its wide and successful application, the resistance of the hydrogel surfaces to cell adhesion and differentiation might represent a considerable limitation. In this context, the hydrogel functionalization, through the incorporation of adhesive molecules, emerges as a promising approach to overcome these limitations.

Several molecules, namely proteins of the ECM (extra-cellular matrix), poly-L-lysine (PLL) and a natural adhesive protein extracted from mussel (MAP) [8] have been successfully applied in promoting cell adhesion and proliferation [8-12]. In addition, the Arg-Gly-Asp (RGD) motif – found in ECM proteins and in the blood, such as fibronectin, vitronectin, osteopontin, collagens, thrombospondin, fibrinogen, and von Willebrand factor – was described as the major functional group responsible for cellular adhesion [8,13,14]. Various strategies of surface functionalization, which include the coupling or grafting of the RGD peptide, have been already reported. Most of these involves complex chemical reactions, to activate the chemical groups in the polymer or in the RGD containing sequence, to allow for the covalent binding [15-17]. In this study, a new approach of RGD-activation of dextrin-hydrogel (a depolymerized starch) is proposed. It has been reported that RGD bioactivity can be conserved in fusion proteins [2,18]. Likewise, a recombinant protein containing a starch-binding module (SBM) and a RGD sequence was used in this work. Several enzymes that metabolize carbohydrates have a modular structure with two independent domains, a catalytic domain and a substrate-binding domain, generically designated as carbohydrate-binding module (CBM). A CBM is defined as a contiguous amino acid sequence within a carbohydrate-active enzyme with a discreet fold having carbohydrate-binding activity. The CBM used in this work, is a starch-binding module (SBM), belonging to a α-amylase from *Bacillus *sp. strain TS-23 [19], which specifically binds to starch [20].

The present work shows the successful functionalization of a dextrin-based hydrogel, using a fusion protein containing a C-terminal SBM and a N-terminal RGD sequence. Viability and microscopic evaluation of the protein-activated hydrogels, revealed an effective improvement of cellular adhesion and spreading.

## Methods

### Reagents and strains

All reagents used were laboratory grade reagents from Sigma-Aldrich, St. Louis, USA, unless stated otherwise.

The bacterial hosts used for cloning and expression of the fusion proteins were *Escherichia coli *strain XL1 Blue [recA1 endA1 gyrA96 thi-1 hsdR17 supE44 relA1 lac [F proAB lacIqZΔM15 Tn10 (Tetr)] (Stratagene) and strain BL21 (DE3) [F- ompT hsdSB (r-B mB-) gal dem Δ(srl-recA) 306::Tn10(DE3)](Novagen), respectively. The pET 29a(+) (Novagen) was used as expression vector. The restriction enzymes and T4 DNA ligase were purchased from Roche Diagnostics GmbH (Penzberg, Germany). The Vent DNA polymerase used was from New England Biolabs.

In vitro assays were performed using mouse embryo fibroblasts 3T3 (ATCC CCL-164), grown in Dulbecco's modified Eagle's media (DMEM) supplemented with 10% newborn calf serum (Invitrogen) and penicillin/streptomycin (1 μg/mL) (Sigma-Aldrich, St. Louis, USA), at 37°C, in a fully modified air containing 5% CO_2_.

### Gene cloning

The DNA coding sequence of the starch-binding module from *Bacillus sp*. Strain TS-23 was synthesized (Epoch Biolabs, Missouri City, USA). This sequence was used as template to clone SBM and RGD-SBM codifying sequences by PCR. In the case of RGD-SBM peptide, 10 amino acids were cloned in the N-terminal between SBM and RGD, to act as a linker and allow some mobility of the RGD. Briefly, SBM and RGD-SBM sequences were cloned using the forward primers 5'-GGGAATTCCATATGACGTCAAACGTCACATTTAC-3' and 5'-GGGAATTCCATATG**AGAGGTGAT**GGAGGCTCCGTTTCGATTTGG-3', respectively, and the reverse primer 5'-CCGCTCGAGTGGCACATTCCAGCTCGC-3', the underline sequences are the restriction sites for the *Nde *I and *Xho *I, and the bold sequence codify the RGD. The PCR reactions were performed using the Vent DNA polymerase and the PCR conditions were: denaturation at 95°C, annealing at 53°C and extension at 72°C, all steps for 45 seconds (this cycle was repeated 30 times).

Both DNA coding sequences were cloned in pET29a(+) expression system, which allows the fusion of recombinant proteins with a hexa-histidine tag on the C-terminal, for purification. The nucleotide sequences of cloned genes were verified by sequencing. The *E. coli *XL1 Blue was used as cloning strain and expression was carried out in *E. coli *BL21 (DE3).

### Production and purification of recombinant proteins

For the production of the recombinant proteins, the *E. coli *BL21 (DE3) cells transformed with the expression vectors, pET29a(+)-SBM and pET21a-RGD-SBD were grown at 37°C, in LB medium supplemented with Kanamycin (50 μg/ml). Cultures were induced with IsoPropyl β-D-1-ThioGalactopyranoside (IPTG, Invitrogen) at 1 mM. Five hours after induction, the cells were separated from the culture medium by centrifugation (13 000 g, 10 min) and resuspended in buffer A (20 mM Tris, 20 mM NaCl, 5 mM CaCl_2_, pH 7.4 and PMSF 0.1 mM) and then lysed by sonication. The soluble and insoluble fractions were separated by centrifugation (15 000 g, 4°C, 30 min). The purification was made by affinity chromatography, using a HisTrapTM HP (GE health care). For that, imidazole was added to the cell lysated (40 mM final concentration) and the pH was adjusted to 7.4 before its application on the nickel column. After purification, proteins were dialyzed against the buffer A, sterilized by filtration (0.22 μm) and stored at -20°C, prior to use.

### SBM adsorptions assays

To evaluate the SBM starch affinity and specificity, an adsorption assay using starch (positive control) and cellulose (negative control). The protein of the soluble fraction (0.5 mL) obtained from the cells lyses (0.5 mg/mL) was mixed with 50 mg of starch or cellulose, for 1 h, at 4°C. Then, the mixture was centrifuged (13 000 rpm, 10 min, 4°C) and the total protein in supernatant was quantified by the Bradford assay (BioRad), using BSA as standards, and analysed by SDS-PAGE. The recombinant SBM was eluted from starch with 2% β-cyclodextrin solution (0.5 mL, 4°C, 1 h).

### Effect of the recombinant proteins on the adhesion and spreading of fibroblasts on the tissue culture polystyrene plate (TCPP)

The cell viability was determined by the (3-(4,5-dimethylthiazol-2-yl)-5-(3-carboxymethoxyphenyl)-2-(4-sulfophenyl)-2H-tetrazolium) (MTS, Promega) assay, a colorimetric assay that gives a measure of the mitochondrial metabolic activity. The fusion proteins were added to the 96-well TCPP (0.05 μg of protein per well) to allow adsorption (4°C, overnight). The unbound protein was washed out with phosphate buffer saline PBS; then 200 μL of fibroblast suspension was plated in each well, yielding a final density of 5 × 10^3 ^cells. After 1 h the wells were washed with PBS and the culture medium refreshed. The MTS assay and microscope observations of the attachment and spreading of fibroblasts were carried out at 1, 5, 24 and 48 h after the addition of the cells.

### Effect of the recombinant proteins on the adhesion and spreading of fibroblasts on the dextrin-based hydrogel

In a second test, the recombinant proteins were added to the hydrogel. For this propose hydrogels were prepared in a 96-well polypropylene plate (autoclavable), as described by Carvalho et al. [3]. Briefly, 30 μL of the dextrin-based solution (300 mg/mL in PBS) was placed on the bottom of each well and the initiators were added to allow the polymerization. After sterilization (20 min, at 121°C and 1 atm) the hydrogel was washed with PBS and then the recombinant proteins were added (0.25 μg of protein per well). Afterwards, plates were incubated overnight at 4°C. Unbound protein was removed and analyzed by SDS – PAGE and the hydrogel was washed out with PBS before cells seeding. A fibroblasts suspension was plated into each well, to yield a final density of 2 × 10^4 ^cells. The plates were incubated and after 4 h, the wells were washed with PBS and the medium refreshed. The MTS assays were carried out on the non-adherent cells. Microscope observations of the attachment and spreading of 3T3 fibroblasts was carried out at 4, 24 and 48 h after the addition of the cells and then trypsinized from the hydrogel before MTS analysis. The results expressed as cell proliferation inhibition index (CPII) were calculated as CPII = 100 - (DO_490 nm _of test culture/DO_490 nm _of control culture) × 100.

## Results and discussion

### Expression and purification of recombinant proteins

**Figure 1:**
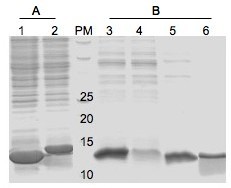
**Analysis of protein expression (A) and starch specificity (B) by SDS-PAGE**. A-Soluble protein extract obtained from lyses of *E. coli *BL 21(DE3) carried pET29a(+)-SBD (1) and pET29a(+)-RGD-SBD (2) vectors. B-Total soluble protein extract (containing SBM) used in adsorption assays (3); supernatant obtained after starch (4) and cellulose adsorption (5), supernatant obtained after protein elution of starch with β-cyclodextrin (6). (MW-molecular weight, KDa).

As shown in figure 1, the SBM and RGD-SBM recombinant proteins were successfully expressed using the pET29a(+) expression system and *E. coli *BL21 (DE3) cells. The proteins were presented in the soluble fraction, exhibiting the expected MW, 12.0 and 13.3 KDa respectively. Previous works used another expression system (pQE, Quiagen) and host (*E. coli *M15) to produce recombinant proteins fused to this SBM [21-23]. However, in the present work the amount of the recombinant protein obtained in the soluble fraction was much higher (40%), indicating that this expression system is preferable.

The SBM functionality was analysed through a starch adsorption assay, confirming that the binding module is functional. The SBM specificity was evaluated using starch (positive control) and cellulose (negative control). The SBM adsorbed only to starch and it could be eluted using soluble β-cyclodextrin (Figure 1).

### Attachment of fibroblasts to the recombinant protein-coated TCPP

**Figure 2:**
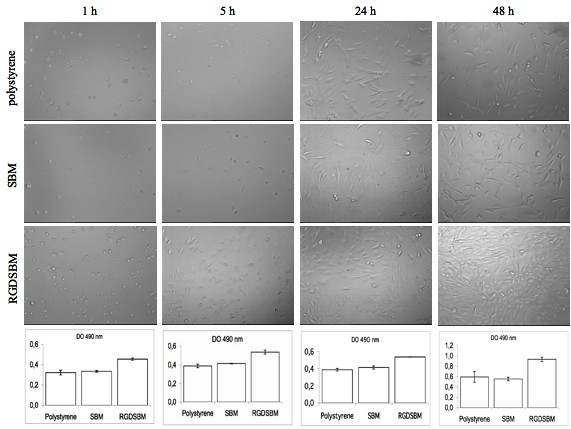
**Microscopic observation and MTS analysis**. Microscopic observation and MTS analysis of the cells attached to the polystyrene plate and polystyrene plate coated with SBM or RGDSBM peptides, at different times (MTS results were performed in triplicate). The MTS assay shows the optical density at 490 nm obtained in different conditions tested.

The TCPP were used as a first approach to observe the effect of the presence of protein coating the material on the adhesion of cells. Thus, both polystyrene and fibroblasts are here considered as a model system. The actual applications envisaged involve the use of dextrin made materials (as described ahead) and other cell lines (not in the scope of this work). The microscopic analysis and MTS results showed that, when the polystyrene plate was coated with RGD-SBM, fibroblast adhesion was improved, as compared to the uncoated wells or the ones treated with SBM (controls). In fact, after 1 h adhesion, the MTS results showed that fewer cells adsorb to the plate coated with SBM, as compared to the ones coated with RGD-SBM (figure 2). Furthermore, the wells with or without SBM adsorbed exhibited the same amount of adherent cells. It seems, thus, reasonable to conclude that SBM did not affect cell adhesion, being the RGD sequence the responsible for the improvement of the CPII. Indeed, a decrease in CPII of about 30 to 35% was achieved in the presence of the RGD-SBM, the same trend being also observed in the assays carried out for longer periods of times (5, 24, 48 h). Regarding morphology, the cells cultivated in different conditions were similar.

### Attachment of fibroblasts on dextrin-based hydrogel surface

**Figure 3:**
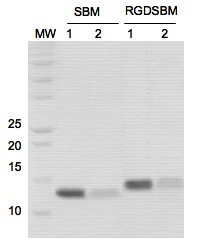
**SDS-PAGE analysis**. Analysis of the recombinant proteins adsorbed to the dextrin-based hydrogel, by SDS-PAGE. Recombinant proteins SBM and RGD-SBM, purified by affinity chromatography, before (1) and after (2) adsorption on the hydrogel. (MW-molecular weight, KDa).

The recombinant proteins were added to the hydrogel and left adsorbing overnight. Before the addition of cells, the hydrogel was washed out with PBS, in order to remove the unbound proteins. The supernatant containing the unbound proteins was analyzed by SDS-PAGE (figure 3), confirming that the SBM successfully adsorbs to the hydrogel and the amount of recombinant proteins used was enough as to saturate the hydrogel. A TCPP was used as a control, for comparison of cell adhesion and morphology. The specificity and stability of the adhesive proteins present in the surface of the biomaterial have been referred as a critical factor for the cells attachment and behaviour [24]. The SBM used in this study belongs to the α-amylase secreted by the *Bacillus *sp. TS-23, a thermophilic and alcaliphilic bacteria [20]. This enzyme is functional under extreme conditions, and it was described as rather stable in a large range of temperatures and pH. Furthermore, it should be remarked that CBM's have been used in the development of commercial systems for recombinant protein purification [25]. Therefore, the adsorption of the SBM on the hydrogel surface may be expected to be stable and specific, allowing its successful application to functionalize the hydrogel surface.

**Figure 4:**
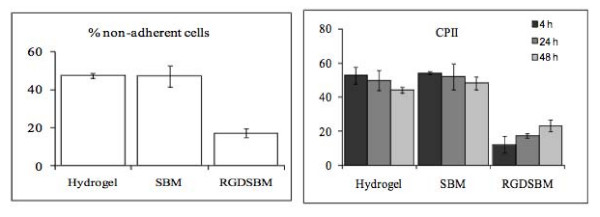
**MTS analysis and CPII determination**. MTS assays from non-adherent cells to the hydrogel and hydrogel coated with recombinant proteins after 4 h of adhesion. CPII of hydrogel with different treatments when compared with the polystyrene plate, at 4, 24 and 48 h of incubation after fibroblasts addition.

The fibroblasts were added to the hydrogel treated with SBM or RGD-SBM and incubated for 4 h (polystyrene and non treated hydrogel were used as controls). Then, the non-adherent cells were washed out and fresh medium was added. To evaluate the cellular adhesion to the hydrogel, MTS assays were performed, both with the non-adherent cells and the ones trypsinized from the hydrogel. The results obtained using the two cell samples (adherent; non adherent) are in good agreement. The MTS results showed that, in the case of the polystyrene, 100% of the cells adhered after 4 h of incubation. In the case of the hydrogel coated with RGD-SBM, about 80% of the total cells were present in the adherent fraction and only 50% of the cells adhered to the hydrogel controls (untreated hydrogel or containing SBM peptide) (figure 4). These results demonstrated that the RGD sequence was able to significantly increase the adhesion of fibroblasts to the hydrogel surface, as compared to the controls. Previous work reported that the CPII of the starch-based hydrogels were in the range 50%–60% when compared to polystyrene [26]. Herein, the same range of values was obtained for hydrogel controls. Nevertheless, when the hydrogel was treated with RGD-SBM, it was possible to reduce the CPII to 17%, which represents an improvement on cell adhesion of more than 30% (the values reported are the average of 2 different assays, each one performed in triplicate).

**Figure 5:**
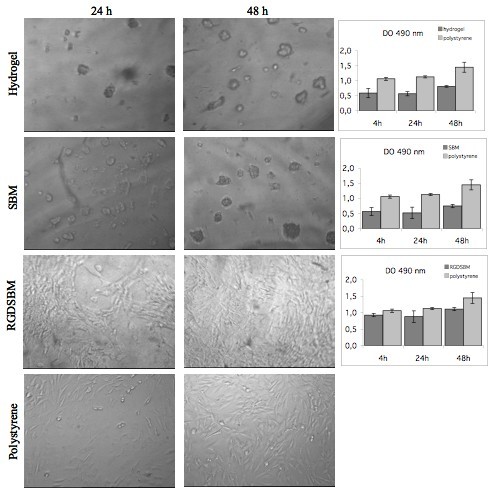
**Microscopic analysis and MTS assays**. Microscopic analysis and MTS assays of the fibroblasts cultivated on hydrogel; without recombinant proteins, coated with SBM or RGDSBM and to the polystyrene plate, at different incubation times. The MTS assay compares the optical density at 490 nm between hydrogel with the different pre-treatments and the polystyrene plate at 4, 24 and 48 h of incubation with fibroblast.

The ability of the biomaterials to promote cell attachment is an important factor for tissue engineering applications. However, other important factors for the survival of the cells must be considered, namely cell spreading, migration, proliferation and matrix proteins production [12,27]. The cellular spreading on the hydrogel was evaluated by microscopic observation. Previous studies on starch-based hydrogels, have shown differences in the morphology of the cells growing on the hydrogel surface [17,28]. Although viable, cells appear rounded and clustered when grown in the hydrogels. In contrast, in the presence of RGD sequence the cells are uniformly distributed on the hydrogel, exhibiting the characteristic fibroblast morphology (figure 5). The cellular spreading was only achieved under these conditions and the confluence was reached after 48 h of incubation.

The effect of the RGD-SBM peptide on proliferation was also evaluated. The MTS results suggested that the cell proliferation was only moderate irrespective of the hydrogel activation, as can be seen by the evolution of the non-normalized absorbance values (figure 5). These results are in good agreement with previous reports [26,29] of a moderate cell growth on similar non-activated hydrogels. It could be expected that the presence of RGD-SBM peptide would increase the proliferation rate. However, despite the relevant effect on cell morphology, the presence of the RGD-SBM does not lead to a significant increase in the rate of proliferation. It is well known that the fibroblast migration and proliferation on a biomaterial surface, coated with adhesive peptides, is dependent on the peptides density [10,12,24]. Neff at al. [24] found that fibroblast migration and proliferation decreased with the increasing of the adhesion peptide concentration. These authors found that for a maximum fibroblast proliferation, the adhesive peptide density should be intermediate. A similar effect was observed for other cells lines, namely, murine melanoma cells [30] and smooth muscle cells [31]. In this work, the effect of the RGD-SBM concentration on both cells migration and proliferation was not evaluated. Likewise, it is possible that the peptide concentration should be under or over the optimum, which would explain the proliferation rate observed. Thus, future work should address the optimization of the peptide concentration in order to maximize cell proliferation on the dextrin-based hydrogel [12].

## Conclusion

In this work, a new approach was applied to functionalize a dextrin-based hydrogel: a recombinant protein, with a C-terminal starch-binding module and a N-terminal RGD sequence was cloned, expressed and successfully used to improve fibroblast adhesion and spreading on the hydrogel surface. The recombinant DNA techniques allow the fusion of different peptides in order to obtain chimeric proteins with specific functionality. However, the fusion of two peptides individually functional does not necessarily lead to a bi-functional fusion protein, according to our own experience. The loss of peptide functionally in the recombinant fusion protein may be a result of conformational changes that interfere with substrate accessibility or cell interaction. This is not the case with the protein produced in this work. The RGD-SBM recombinant protein improved by more than 30% the adhesion and spreading of fibroblast on the starch-based hydrogel.

The major advantages of the approach developed in this work may be summarized as follows: 1) the RGD sequence is expressed in *E. coli *fused to the SBM, not chemically synthesized; normally, the RGD sequence obtained chemically is attached to a linker and/or a reactive amino acids, and thus are expensive products. 2) The fusion protein strongly and specifically binds dextrin or starch-based materials, without the need for complex chemistry, toxic chemicals, etc. The specific affinity of the SBM may be used also for the purification of the protein. 3) This approach, here demonstrated with the RGD case study, may be adopted to a wide range of peptides, particularly to short peptides like SBM, which may easily be produced using this expression system. 4) Several applications may be envisaged for this system: the production of biomimetic materials for the development of cell culture medium, the functionalization of materials for cell immobilization or even biomedical applications (addressing multifunctional nanoparticles made of starch to neoplastic tissue by RGD active targeting, for instance). Preliminary results show that the foreign body reaction to hydrogel implants is not affected by the presence of RGD-SBM.

## Authors' contributions

SM carried out the gene cloning, recombinant proteins expression and purification, 'in vitro' studies and drafted the manuscript. FA participated in 'in vitro' assays. LD participated in the revision of the manuscript. FG conceived the study, participated in its design and coordination and helped drafting the manuscript. All authors read and approved the final manuscript.
